# Supplementary material for: Knowledge, attitudes, and practices on anthrax in selected game management areas in Zambia
Source: PLoS Negl Trop Dis. 2025 Sep 30;19(9):e0013592. doi: 10.1371/journal.pntd.0013592 (PMC12503290; doi:10.1371/journal.pntd.0013592)
Supplement: Attachment — Submitted filename: Response to Reviewers-PloSNTD 28-7-25.docx [file pntd.0013592.s002.docx]

**Response to Reviewers – Revised Manuscript Submission**

***Manuscript Title: Knowledge, Attitudes, and Practices on Anthrax in Selected Game Management Areas in Zambia***

Dear Editors and Reviewers,

We sincerely thank you for your thoughtful and constructive comments on our manuscript. We have carefully considered all feedback and made substantial revisions to strengthen the clarity, rigor, and overall quality of the paper. Below, we provide a detailed, point-by-point response to each of the reviewers’ suggestions and concerns. All changes made are highlighted in the revised manuscript.

**Reviewer #1**

**Comment:**
The methods were well described with details on the study location, design, and sampling. However, there is a discrepancy in the sample size between lines 146 and 147, where it is stated that the sample size was 1,155, but after removing incomplete questionnaires, the final sample size is noted as 1,185, which is higher.

**Response:**
Thank you for identifying this inconsistency. We have corrected and clarified the paragraph in the manuscript. The revised sentence in the Results section now reads:

*“We initially targeted 1,155 participants based on sample size calculations; however, we received 1,193 responses and retained 1,187 after removing incomplete questionnaires.”*

**Reviewer #2**

**Comment:**
The methods section needs to be strengthened. Please explain the rationale for the sample size, the use of a 50% estimate, and the design effect of 3.

**Response:**
We appreciate this observation. The revised manuscript now includes a clearer explanation of the sample size calculation formula, assumptions (50% prevalence, 95% confidence level, 5% margin of error), and the design effect of 3. This has been placed under the Sample Size Estimation subsection.

**Comment:**
Why did the authors not use a sample size formula that accounts for clustering?

**Response:**
Thank you for this important point. Although the study covered distinct geographic areas, we employed stratified random sampling without applying a clustering adjustment. This decision was based on the independent administration of the survey across sites, and no hierarchical nesting was observed in the data structure.

**Comment:**
The unit of analysis should be clearly stated.

**Response:**
This has now been clarified in the manuscript. The unit of analysis was the *individual household respondent*.

**Comment:**
There is no clear description of the sampling and recruitment process. Also, how was clustering taken into account in identifying participants?

**Response:**
We have revised the Data Collection section to include the following clarification:

*“Participants were recruited through community mobilization by local leaders and extension officers. Each selected village was visited, and households were randomly approached until the target number was reached. Enumerators obtained verbal consent and conducted face-to-face interviews using structured questionnaires.”*
Clustering was not applied during analysis for the reasons outlined earlier.

**Comment:**
The analysis section is lacking detail, including different analysis types and rationale, model-building processes, outcomes, inclusion criteria, and significance thresholds.

**Response:**
Thank you for this valuable feedback. We have substantially revised the Data Management and Analysis section to include:

- Detailed scoring approaches for Knowledge (binary), Attitudes, and Practices (Likert scale and quartile grouping)
- Statistical tests used (Chi-square, Kruskal–Wallis, Spearman’s rho)
- Model-building: multivariable logistic regression with variables included based on p < 0.20 from bivariate analysis, using backward stepwise elimination
- Clear reporting of adjusted odds ratios (AOR), confidence intervals, and model fit (Hosmer–Lemeshow test)

**Reviewer #3**

**Comment:**
Did you collaborate with anthropologists when developing the questionnaires?

**Response:**
Yes, the research team was multidisciplinary, comprising anthropologists, animal health economists, ecologists, epidemiologists, veterinarians, gender specialists, and others. While we did not list individual disciplines in the manuscript, the tool development process benefited from this wide expertise.

**Comment:**
Please elaborate on the use of logistic regression.

**Response:**
This has now been addressed comprehensively in the revised Data Management and Analysis section. Five paragraphs have been added to describe the rationale, process, and interpretation of the logistic regression model used.

**Comment:**
Were the surveys administered online? If so, this should be mentioned in the Limitations section.

**Response:**
Thank you for this clarification request. The surveys were conducted in-person using electronic devices (tablets and mobile phones). They were not self-administered online. We have updated the manuscript to reflect this and have included a note in the Study Limitations section regarding the potential limitations of digital data collection in remote settings.

**Results Section**

**Reviewer #1:**
The results were well described and presented in appropriate tables.

**Response:**
Thank you for the positive feedback.

**Reviewer #2**

**Comment:**
There is a mismatch between the Methods and Results sections, and some results (e.g., logistic regression) are only discussed later.

**Response:**
Thank you for the observation. We have restructured the manuscript to ensure that all results are clearly described under the Results section and correspond to the methods described. This includes moving KR-20 internal consistency test results from Methods to Results.

**Comment:**
Sociodemographic data should be presented by site, and completeness of data should be reported.

**Response:**
We have maintained overall sociodemographic presentation to facilitate statistical analysis of associations. However, data disaggregated by site have been published in a related earlier paper:

<https://journals.plos.org/plosntds/article?id=10.1371/journal.pntd.0012893>

**Comment:**
Table 8’s title should better describe the model, and the meaning of asterisks should be explained.

**Response:**
We have revised the title to:

*“Table 7: Multivariable logistic regression model identifying predictors of poor anthrax knowledge (score <60%) among respondents”*
Footnotes (key) have been added to explain the asterisks:
*p < 0.05, **p < 0.01, ***p < 0.001*

**Comment:**
How was poor knowledge defined? Were all variables in the model?

**Response:**
Poor knowledge was defined as a score below 60%, in line with Bloom’s cut-off. We have clarified that variables with p < 0.20 from bivariate analysis were entered into the model, and backward stepwise elimination was applied. Only statistically significant variables were retained in the final model, and model fit was assessed.

**Comment:**
Figure 3 lacks a description.

**Response:**
We have added a detailed description of Figure 3 in the Results section, explaining what the figure shows, including age group comparisons, the impact of education, and interpretation.

**Editorial and Structural Revisions**

**Reviewer #1:**
Recommended minor revision.

**Response:**
Thank you for this recommendation. We have implemented all suggested corrections and improvements.

**Reviewer #2:**
Tables and figures would benefit from more descriptive titles and footnotes.

**Response:**
We agree and have revised all table and figure captions to be more descriptive, and added explanatory footnotes where appropriate.

**Reviewer #3:**
Recommended that the Sampling Strategy section be moved earlier, and results moved out of the Methods section.

**Response:**
These changes have been made. The Sampling Strategy section now precedes the Study Sites section, and all results have been moved to their appropriate section.

**Summary Comments**

**Reviewer #1:**
The paper is well-structured and well-written apart from minor issues.

**Response:**
Thank you for your kind comments and helpful suggestions.

**Reviewer #2:**
The study is interesting, but more detail was needed in the Methods and Results to strengthen interpretation and impact.

**Response:**
We are grateful for this constructive feedback, which has helped us significantly improve the clarity and robustness of the manuscript.

**Comment:**
The link between anthrax and wildlife trade should be better explained.

**Response:**
We appreciate this point. We have clarified that anthrax is not transmitted through live animal trade, but rather through illegal meat trade involving the handling and consumption of infected carcasses—particularly in areas where wildlife carcasses are accessed and butchered.

**Reviewer #3:**
The article is interesting with rigorous methods. Just a few structural adjustments were needed.

**Response:**
Thank you for your encouraging feedback. We have implemented all structural suggestions accordingly.

**Final Note**

We are deeply grateful to the reviewers and editors for their insightful comments and suggestions, which have significantly improved the quality of our manuscript. We hope the revised version meets your expectations and look forward to your favorable consideration.

Sincerely,

**Dr. Chisoni Mumba**
*On behalf of all co-authors*
